# Environment and cancer: the legacy of Lorenzo Tomatis

**DOI:** 10.1186/1476-069X-10-S1-S1

**Published:** 2011-04-05

**Authors:** Rodolfo Saracci, Paolo Vineis

**Affiliations:** 1 IFC- National Research Council,via Trieste 41, 56126 Pisa, Italy; 2 MRC/HPA Centre for Environment and Health, Imperial College, St.Mary’s Campus, Norfolk Place W2 1PG, London, UK

## Abstract

As an introduction to the series of papers arising from the first ‘Lorenzo Tomatis Conference on Environment and Cancer’ , Tomatis’ contributions to research in cancer prevention are first noted, especially the ongoing programme ‘IARC Monographs on Carcinogenic Risk for humans’ that he established at the International Agency for Research on Cancer’ , of which he was the Director from 1982 to 1993. The programme, started in 1972, has become an international authoritative reference and represents an early ‘evidence-based’ development bringing together a comprehensive evaluation of both experimental and epidemiological data. Next the recurrent issue of how large is the contribution of environmental factors to cancer etiology is examined pointing to the several limitations making estimates of the population fraction of cancers attributable to environment delicate to interpret or sometimes even misleading. Finally mention is made of societal issues such as social inequalities in cancer occurrence and fatality, communication in the clinical oncology and cancer prevention and screening areas and the relation between these and the blossoming basic cancer research boosted by the revolution in molecular biology and genetics.

## 

The present supplement to *Environmental Health* is based on a meeting held in 2009 in Torino to remember Lorenzo Tomatis’ extraordinary contribution to science and Public Health. In this introduction we want to mention just a few of the open issues in environmental epidemiology today, to which Tomatis’ legacy has still much to offer.

As he noticed several times in the past, we are often confronted with two opposite attitudes towards prevention, that are well reflected in the conundrums affecting the application of the 'precautionary principle'.

On one side, we have scientists who only believe in ‘hard’ science and are reluctant to accept evidence that falls short of a rigorous experimental demonstration. This is exemplified by the attitude of many scientists in the ‘50s who did not accept the evidence, often decried as ‘only statistical’, on the adverse health effects of tobacco smoking in the absence of randomized trials in humans and supporting evidence in animal experiments.

On the other hand, we have environmental associations (as the International Society of doctors for Environment –ISDE- of which Tomatis was a founder ) that are deeply involved in primary prevention, often within a network of committed physicians. These associations base their activities on precaution, strongly holding the view that no exposure to hazardous agents should occur.

As a principle this is all right but it raises the key issue of what we define as an ‘hazard’ , particularly in an increasingly technological world where we are continuously confronted with new man-made agents with unknown biological properties (needless to say the hazard identification is only step one in the sequence leading from aetiology to prevention). First, an operational definition is needed – such as the one introduced by the IARC Monographs on Carcinogenic Risk for Humans [1]. Second, sometimes the definition of hazardous exposure appears affected by a flawed reasoning which roughly runs as follows. Several chronic diseases are rising in frequency ; scientists cannot clearly and quantitatively explain why the increase is occurring; environmental exposures have increased as well and new potential threats are around us (e.g. nanotechnologies) : therefore doubtful or negative investigations that cannot find a relation between environmental exposures and diseases must be wrong and/or affected by the investigators conflicts of interest. As a consequence, action should be taken on the basis of a suspicion, not of reasonably sound evidence. The literature on mobile phones offers an example of the two opposite attitudes : it is interpreted as totally unconvincing by ‘hard-line’ scientists and as sufficient to guide action by activists who suspect that negative findings are simply the reflection of conflicts of interest and/or poor science.

We do not have a ready made solution. We simply want to mention that such problems of Public Health were clearly identified by Tomatis already 40 years ago; and that awareness of the tension between science and the precautionary principle is a good starting point, rather than dogmatically rallying on one of the two extreme views sketched above

## The burden of environmental cancer today

The sum of the effects of identified environmental hazards leads directly to the deceivingly simple question of how large is the burden of cancer attributable to the environment . Strictly speaking 100% of cancers are due to environment if ‘environment’ is defined as e.g. by Higginson in 1979 [2] ‘ ..what surrounds people and impinges upon them…the air you breathe, the culture you live in…the chemicals with which you come in contact’ . Along the same line the ‘Dictionary of epidemiology’ [3] defines environment as ‘ All that which is external to the individual human host’.

No disease , in fact no physiological trait, can develop without the interaction of the host , and of its genetic endowment, with the surrounding environment. In this sense all diseases have necessarily an environmental causal component. This makes clear that the reply to the question of what proportion of cancers are due to environment depends in the first place on how broad or restrictive definition of environment one chooses, any such choice being based on scientific as well as subjective considerations, often value-laden. For instance should tobacco smoking be included under the heading of ‘environment’ ? No doubt tobacco smoke is an agent present in the personal or (micro) environment of an individual ; however while everyone would agree that environmental tobacco smoke due to ‘passive’ smoking qualifies for inclusion, some would argue that tobacco smoke from active smoking should be excluded from ‘environment’ and classified with lifestyle factors or personal habits. Thus there can be ample room for unintended or intended confusion in the exercise of trying to apportion the contribution of environment to the aetiology of cancers.

One telling example is the alleged gross overestimation of 19 % produced by a WHO headquarters team (Prüss-Üstün and Corvalan, [4]) when compared to an estimate roughly ‘ one order of magnitude lower’ deemed by Boffetta et.al [5] in line with authoritative references such as the widely quoted Doll and Peto estimate [6]. In fact the WHO team used a broad definition of environment and Doll and Peto a narrower one including only air, water and soil pollutants. When the latter [7] was applied to the WHO team data the discrepancy vanished : the WHO estimate reduced to 5.1% less than twice the Doll and Peto estimate of 3%. If anything these two figures are surprisingly close taking into account that the Doll and Peto estimate is based on USA data prior to 1980 and the WHO team refers to recent data for the whole world.

To derive a measure of the burden of cancers due to agents grouped under the heading of ‘environment’ , namely to compute the ‘Population attributable fraction’(PAF) requires two quantitative elements : first the distribution of all the agents and their levels in the population and, second, the cancer risk associated with each level of exposure to such agents. Apart from the difficulty of obtaining relevant and accurate data (particularly as to the distribution of the agents in the population) it is immediately clear that any estimate of a PAF is population specific and that any ‘overall’ PAF for a population heterogeneous as to the agents distribution can be very misleading. For instance recently De Matteis et al. [8] found that ‘…the PAF [for lung cancer attributable to occupational agents] reported by 32 studies among men vary greatly in time and space : they ranged between 0% and 40% according to different geographical prevalence of hazardous industries’.

Because of this and other critical considerations we discussed in a previous article [7] a PAF figure should be regarded –whatever the sophistication in its calculation- as a pointer to the impact of causal agents on a population rather than as an accurate measurement of a uniformly distributed impact. Certainly in view of the important sources of inaccuracy entering their derivation it does not make sense to report PAF percentages to the second decimal . A more generally realistic scale applicable with the kind of ‘soft’ data usually available would be to simply classify PAF into categories such as : <0.1% ; 0.1% to 2% ; >2 %-10% ; 11%-20%;21%-40%;41%-60%;61%-80%;>80%. Within this frame the PAF attributable to environmental agents , inclusive of tobacco smoke , would fall for a typical economically developed country in the fifth category (21% to 40%). If the full burden from infectious micro-organisms would be included the PAF would move into the sixth category (41% -60%) where it would also stay a ‘worldwide overall’ PAF.

## Evaluation of carcinogenicity of environmental agents

A simple classification of environmental agents into “carcinogenic” or “non carcinogenic”, is an inadequate basis for decision-making, if it is divorced from a complete and critical description of the evidence at hand. Such simplification of messages, as frequently done by the press or, worse, by actors with a vested interest, implies little consideration of the ability of the lay public to understand scientific facts and their interpretation. The IARC Monographs programme [1] ,started in 1972, anticipated by at least a decade one of the most influential changes in medicine, “evidence-based medicine”. By setting rigorous criteria to summarize and evaluate the scientific evidence, the Monographs programme developed by Tomatis brought an evidence-based approach into Public Health.

The IARC Monographs have championed such an approach to the translation of scientific evidence into prevention. Not only rigour in applying causality criteria, but also a full and detailed account of the data at hand, is the only way to help society to address risks for health from occupational and environmental exposures. From this point of view the Monographs have been an instrument for 'deliberative democracy' in Public Health. To become closer to developments in 'evidence-based Public Health' IARC has recently added proper meta-analyses , not included in the original Monographs programme. The concepts and criteria that guide the literature summary and evaluation remain the same as in the past but they are strengthened by the application of formal meta-analytical tools.

## The contributions in the present issue

It was not the purpose of the conference to carry a comprehensive review of cancer prevention in its research and applied aspects. Rather the contributions, most of them presented in synthetic form in this issue, focused on themes on which Tomatis was himself involved all along his professional life. The address by C.Wild , Director of IARC [9], on the planned directions of work of the Agency highlighted a long lasting fidelity to the scientific and public health mission of the IARC that owes much to Tomatis inspirational initiatives.

The concluding round-table of the conference on ‘Cancer and Society’ touched on several issues, three of them receiving special emphasis. First attention was drawn on social inequalities in cancer occurrence and fatality not only between economically underdeveloped and developed countries but also within countries , where marked social classes differences persist, as does the insufficient recognition of cancers due to workplace exposures.

Secondly the broad issue of communication emerged in its various facets. Within the cancer management area communication of diagnosis and treatment options to patients and families is still very uneven, not seldom frankly inadequate. Education of the public still lags, in general, behind needs in the areas of prevention and early diagnosis. And ,last but not least, messages that the public receives from professionals lack often a balance between encouraging reasonable hopes and avoiding triumphalism as reflected in formulas like ‘ winning the war against cancer’.

Finally the round-table discussion addressed the relation between basic cancer research on one side and prevention and clinical management on the other. The revolution in molecular biology and genetics has now put on a firm factual basis the theory that cancer is a genetic disorder of somatic cells, paving the way to identifying all genetic changes that have taken place between the host genome and the tumor genome and exploring which of these transitions may depend on environmental factors. This all area of investigation is expanding at very fast pace and holds promise of results relevant to preventive and therapeutic practice. A measure of scientific wisdom is however apposite as expressed at the conference by L.Luzzatto : ‘On the other hand , much recent literature gives the impression that there is a surplus of information, from gene expression profiles to proteomics to metabolomics with the risk that while we are truly drowned by data, we remain thirsty of knowledge…..real research , today like before, can only progress by asking questions and formulating testable hypotheses’. Surely Tomatis would have agreed.

## Competing interests

The authors declare that they have no competing financial or non-financial interests.
